# Spilled Gallstones Silent for a Decade: A Case Report and Review of Literature

**DOI:** 10.7759/cureus.2921

**Published:** 2018-07-05

**Authors:** Aisha Akhtar, Marvi M Bukhari, Usman Tariq, Abu Baker Sheikh, Fasih Sami Siddiqui, Muhammad Saad Sohail, Amina Khan

**Affiliations:** 1 Surgery, Texas Tech University Health Sciences Center, Lubbock, USA; 2 Internal Medicine, Shifa College Of Medicine, Islamabad, PAK; 3 Research Assistant, Yale University School of Medicine, New Haven, USA; 4 Internal Medicine, University of New Mexico, Albuquerque, USA; 5 Internal Medicine, Shifa International Hospital, Islamabad, PAK; 6 Internal Medicine, Shifa Tameer E Millat University/shifa International Hospital, Islamabad, PAK

**Keywords:** spilled gallstones, abdominal abscess, laparoscopic cholecystectomy

## Abstract

Laparoscopic cholecystectomy is associated with complications such as gallbladder perforation and spillage of gallstones. While these shortcomings are common, the occurrence of the resultant nuisances, such as intra-abdominal abscesses, is infrequent. We present the case of an individual who developed an intra-abdominal abscess following a spillage of gallstones, which occurred after a laparoscopic cholecystectomy that was performed more than a decade ago. Herein, we also discuss the findings of a literature review that highlights the clinical presentations of an intra-abdominal abscess formed due to gallstone spillage after a decade of the laparoscopic intervention. We also discuss the underlying pathophysiology leading to abscess formation, the imaging modalities used to visualize the abscess, as well as the therapeutic strategy used to treat this rare clinical entity.

## Introduction

Laparoscopic cholecystectomy is the prevailing treatment option for cholelithiasis, and the procedure is associated with low morbidity and mortality. However, the practice of laparoscopy has also led to the advent of difficult complications such as gallbladder perforation and the intraoperative spillage of gallstones. Gallbladder perforations can occur in up to 40% of laparoscopic cholecystectomies while the incidence of gallstone spillage can range from 6%-30% [1]. We present a case and a literature review related to this clinical procedure, characterized by the formation of intra-abdominal abscesses that manifested clinically after more than a decade of undergoing an operative intervention.

## Case presentation

A 78-year-old male with multiple comorbidities, including hypertension, hyperlipidemia, and type II diabetes mellitus, presented to our emergency setting with complaints of recurrent bouts of abdominal pain and fluctuating fevers for the previous two weeks. The patient reported that the pain is a new manifestation of a previously dull aching pain that had waxed and waned over the last decade. His description alluded to a pain that was sharp and intermittent with localization in the right upper quadrant. He could not attribute the intermittent nature of his predicament to any aggravating or relieving influences. The pain was associated with fluctuating low-grade fevers (99°F-100°F), anorexia, and an associated 13-pound weight loss, which culminated in a visit to our clinical setup.

Further interrogation disclosed that the patient underwent a laparoscopic cholecystectomy in 2003. The ensuing year was relatively pain-free but was followed by recurrent bouts of right upper quadrant pain, albeit less upsetting than his current presentation. He was subsequently diagnosed in 2005 with gallstone spillage. The patient chose conservative treatment for his abdominal pain, rather than invasive interventions, which included the administration of acetaminophen and non-steroidal anti-inflammatory drugs (NSAIDs). This treatment modality was sufficient for the duration of a decade. He presented to another medical facility with similar complaints of fever and abdominal pain in 2016. A computed tomography (CT) scan of his abdomen disclosed the presence of a necrotic phlegmon, which was subjected to aspiration. Its composition included a combination of fibrous material, granulation tissue, and inflammatory infiltrate. The aspiration provided considerable relief of symptoms and he was discharged on a gabapentin prescription that was well-tolerated and produced sustained amelioration of his pain, with only occasional wavering with respect to his baseline.

**Figure 1:**
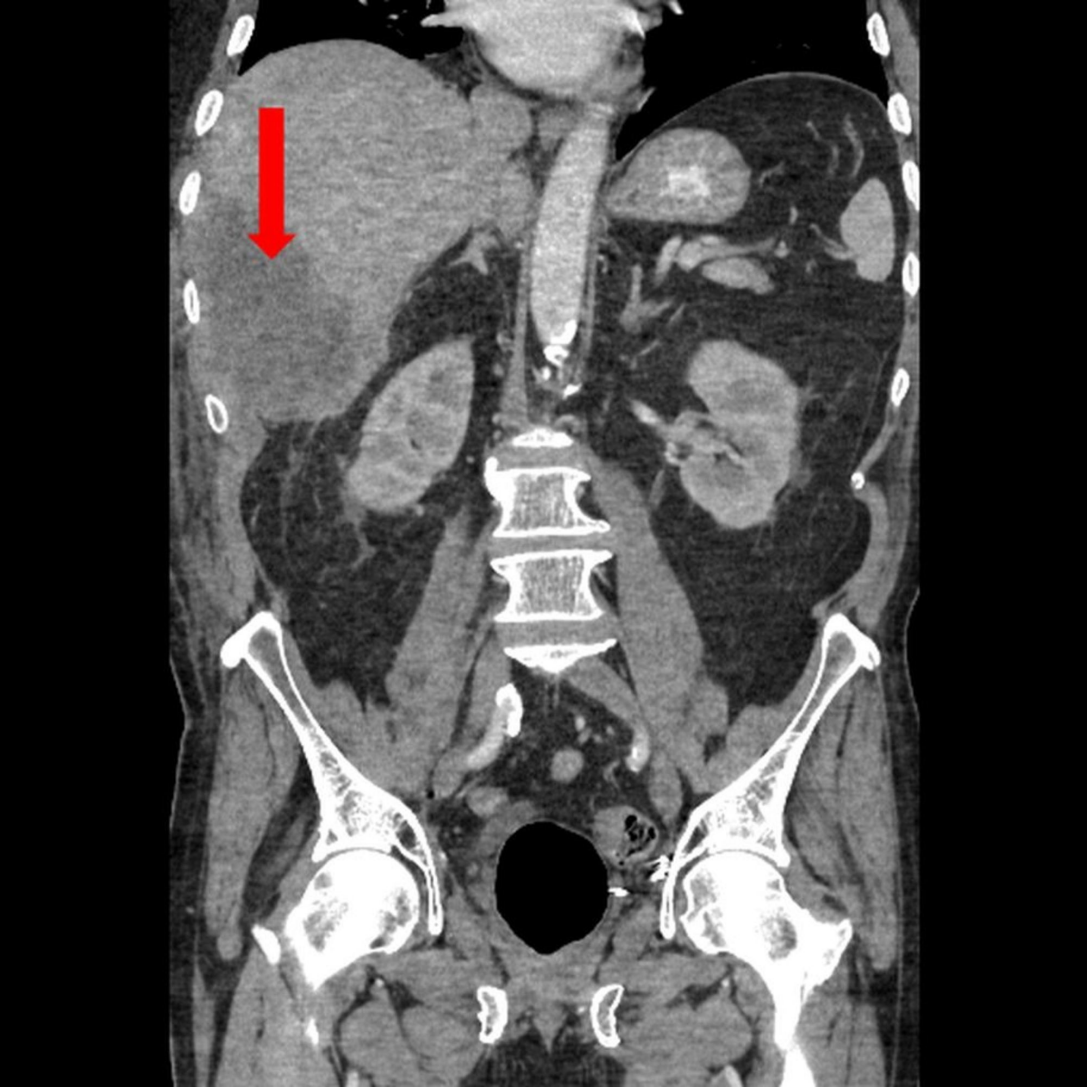
Whole body computed tomography scan showing a large 19-cm sub-diaphragmatic and a right retro-peritoneal abscess inferior and posterior to the right hepatic lobe (red arrow)

The initial assessment showed an elderly gentleman, who was alert and well-orientated but under considerable distress due to the abdominal pain and accompanying chills. He had a fever of 104°F, a heart rate of 120 beats per minute, a blood pressure of 95/70 mm Hg, and a respiratory rate of 19 per minute. An abdominal exam revealed a non-protuberant, soft, and tender abdomen with marked sensitivity in the right upper quadrant. He had perceptible bowel sounds, an absence of dullness on percussion, and a clear rectal vault. Pertinent initial laboratory investigations included an elevated white blood cell (WBC) count of 16,900/µL, C-reactive protein (CRP) of 6.5 mg/dL, and a random blood glucose (RBG) of 280 mg/dL. The patient subsequently underwent a whole body CT scan, which revealed a large 19-cm sub-diaphragmatic, right retroperitoneal abscess, which was inferior and posterior to the right hepatic lobe (Figure 1).

**Figure 2:**
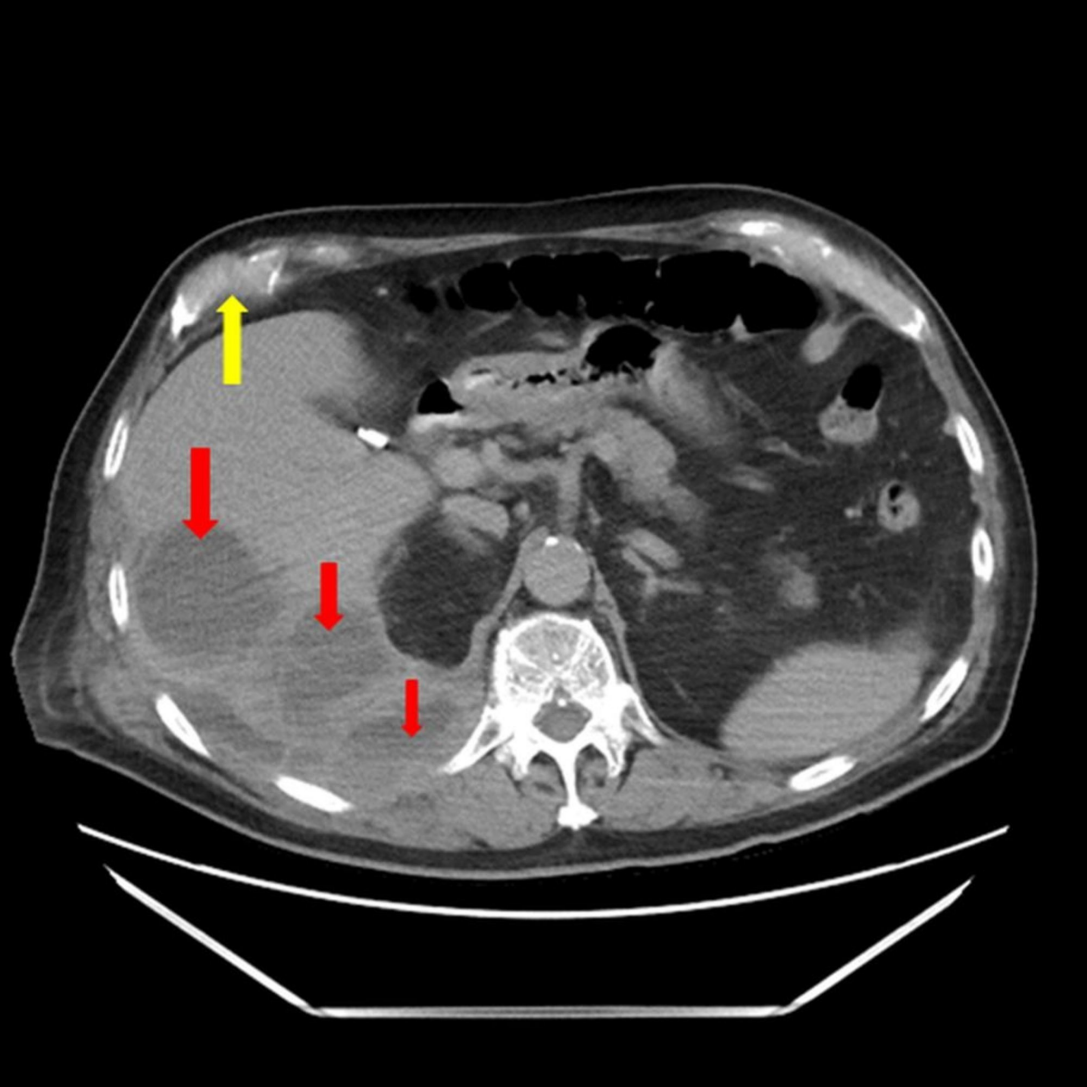
Abdominal computed tomography scan shows a diminished right retro-peritoneal collection inferior and posterior to the right hepatic lobe (red arrows), with a right-sided partially loculated pleural effusion (yellow arrow)

In lieu of these findings, the patient was admitted and started on a combination of intravenous fluids, intravenous (IV) vancomycin and piperacillin-tazobactam due to an underlying suspicion of sepsis secondary to a hepatic and/or perihepatic liver abscess caused by a lack of gallstone retrieval following his cholecystectomy. He was subsequently subjected to a percutaneous CT-guided drain placement, which allowed for the evacuation of approximately 700 mL of grossly purulent material and provided prompt pain relief. The fluid sample was sent for a gram stain, culture, and a bilirubin assay. The gram stain of the abscess fluid revealed branching gram-positive rods concerning for *Actinomyces*, *Nocardia*, and *Streptomyces*; therefore, the patient remained on IV vancomycin and piperacillin-tazobactam, with the addition of trimethoprim-sulfamethoxazole (TMP-SMX) to the antibiotic regimen. Remarkably, the culture growth was positive for *Propionibacterium* and ampicillin-sulbactam was added to the list of antibiotics. Two days later, the patient was shifted to the intensive care unit (ICU) following unrelenting fever and persistent tachycardia, even after his abdominal drain placement was supplemented with vigorous antibiotic and antipyretic therapy. His ICU stay was complicated by episodes of dyspnea, tachypnea, and pleuritic chest pain, while a chest auscultation unveiled a decrease in breath sounds at the right basal region. A repeat CT scan confirmed the reduction in the size of the abscess and showed a new right-sided pleural effusion, which explained the patient’s breathing difficulties (Figure 2).

**Figure 3:**
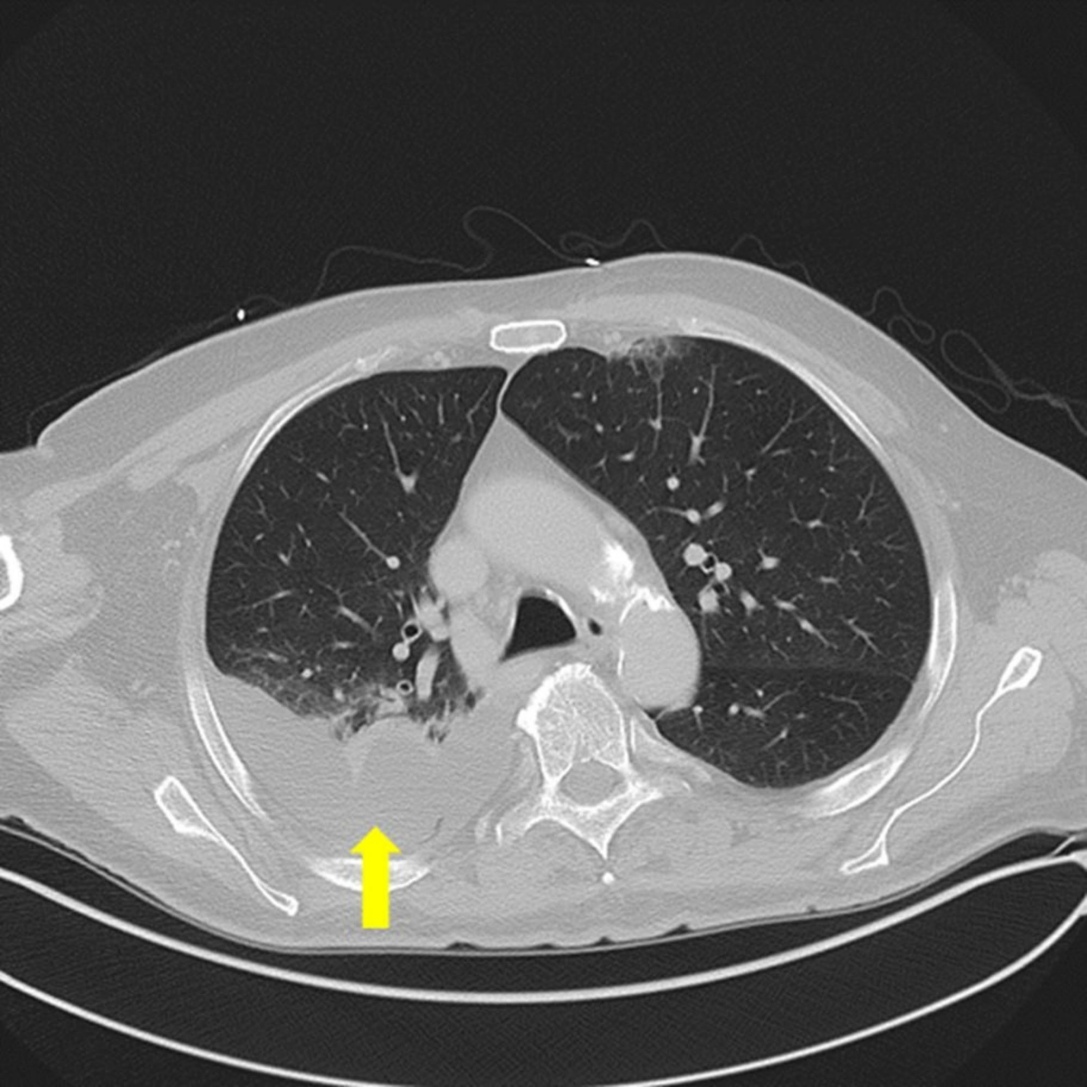
Chest computed tomography scan showing a right-sided basilar opacity with a moderate-sized pleural effusion (yellow arrow)

The patient underwent an ultrasound-guided chest tube placement, which evacuated 10 mL of purulent fluid, followed by two doses of lytic therapy with tissue plasminogen activator (tPA) via the catheter. The following morning, he described an aggravation in his pleuritic chest pain, and an episode of shortness of breath, whereby, the oxygen saturation dipped to 90%. A new chest CT scan was ordered, which showed an interval increase in the right basilar opacity with a moderately sized pleural effusion (Figure 3).

**Figure 4:**
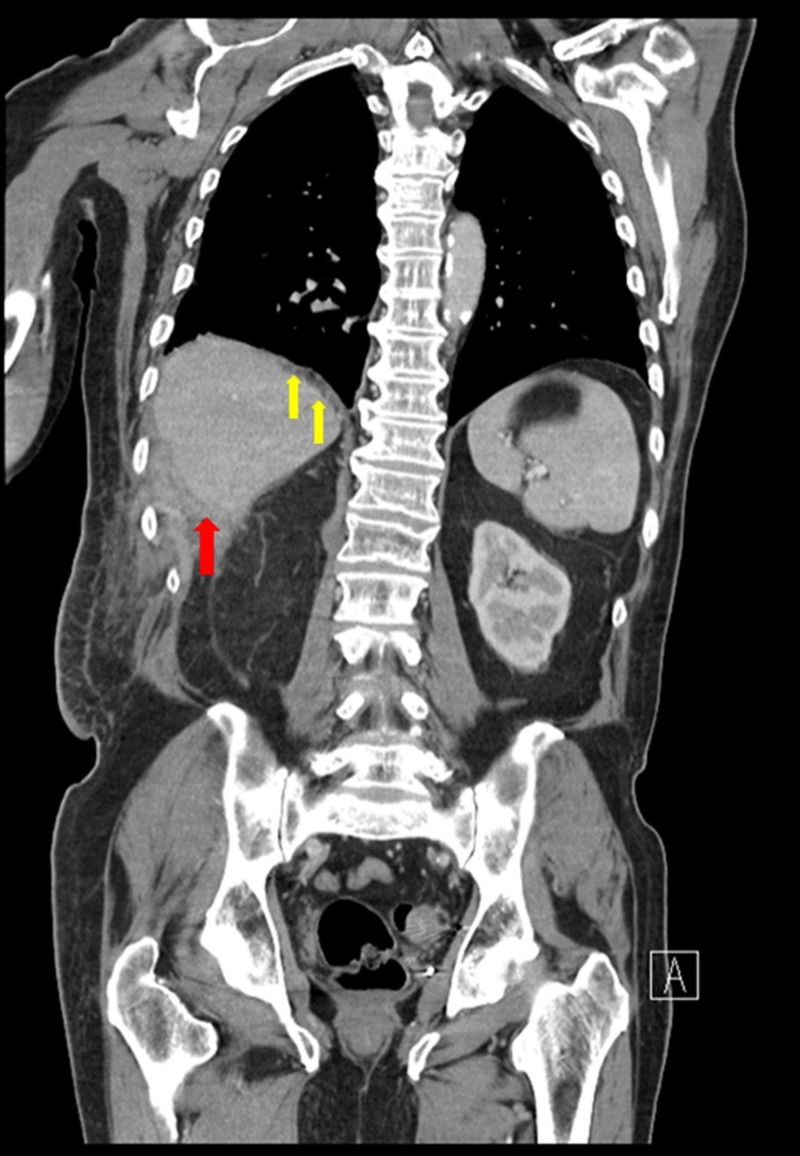
Whole body computed tomography scan showing a diminishing abscess in the inferoposterior aspect of the right hepatic lobe (red arrow) with a diminishing right-sided pleural effusion (yellow arrows).

He was subsequently upgraded to a larger catheter via a CT-guided approach that evacuated a total of 900 mL of purulosanguinos fluid. The patient tolerated subsequent lytic therapies well and showed vast clinical improvement following this procedure, with a reduction in dyspnea, pleuritic chest pain, and fever, as well as a down-trending of his inflammatory markers. A repeat CT scan was performed on the 10th day of admission, which revealed a near-complete resolution of his right-sided pleural effusion, and a decrease in his right-sided retrohepatic intrabdominal abscess (Figure 4).

The chest drain was removed and the patient was subsequently discharged with a transhepatic drainage catheter, daily intravenous ampicillin-sulbactam, and acetaminophen, as needed for pain control. The patient was shifted to oral amoxicillin-clavulanate a month after the resolution of his pain, fever, and stabilization of inflammatory markers. The transhepatic percutaneous drain was left in place in order to allow for the formation of a chronic drainage tract, with hopes of localizing the perpetrating gallstones at its base.

Two months after discharge, the patient underwent a procedure for upsizing the drain in order to accommodate for a choledochoscope into the abscess cavity and the maturing sinus tract. He subsequently underwent an abscess cavity endoscopy, whereby a catheter and scope were advanced into the cavity for exploration. An abscess catheter study was performed through the existing catheter and showed no filling defects, after which the catheter was removed and an endoscope was inserted for direct visualization of the main cavity, as well as the two “fingers” branching out of the abscess cavity. Two small stones were expelled after flushing the main cavity multiple times. Following the expulsion of the gallstones, a replacement catheter was advanced into the cavity and a second catheter study was performed to rule out the possibility of any other stones. The patient tolerated this intervention well and had no further drainage from the intra-abdominal drain.

Following a lack of drain output and resolution of symptoms after his last endoscopic procedure a month ago, the patient's intra-abdominal drain was removed. The patient is currently stable and is being followed as an outpatient to date.

## Discussion

The complication of gallstone spillage following cholecystectomy is mostly affiliated with novel laparoscopic intervention. Papasavas et al. concluded that out of 129 cases of gallstone spillage, the conservative open approach had only two cases of this subsequent complication [2]. An array of various factors subsequently disposes to perforation such as the dissection of the gallbladder from the liver bed, the application of undue retraction of the gallbladder via forceps, as well as gallbladder extraction, which can also potentiate spillage [3].

As illustrated, the risk of perforation and spillage is high [1]. However, the subsequent development of actual complications secondary to a stone spillage is relatively rare, with an estimated incidence of 0.8%-8.5% [3]. This alludes to the understanding that certain risk factors influence the possibility of developing complications. A consensus exists that any factor can conceivably precipitate to infection due to the risk of introducing infective pathogens into the peritoneum. Some of these instigators include advanced age, male gender, a simultaneous existence of acute cholecystitis, and the presence of multiple (>15), sizable (>1.5 cm), or pigmented gallstones [4].

The most common complication of the spillage is the resultant formation of an intra-abdominal and abdominal wall abscess [5]. Pigmented stones and nonsterile bile contain infective pathogens, and the presence of either subsequently enhances the risk of abdominal abscesses [1]. It is for these reasons that most abscesses contain the same flora as that of an infected gallbladder, such as *Escherichia coli* [3]. Our patient presented with an abdominal abscess after a laparoscopic cholecystectomy, which was performed more than 10 years ago. An indolent course such as this can be ascribed to the presence of sterile gallstones within the abdominal cavity, which ultimately manifests as an abscess following the invasion of the bloodstream by a pathological organism. A subsequent infection of the calculus and its adjacent vicinity may lead to a localized inflammatory response, which consequently leads to the formation of an abdominal abscess [6]. Another postulation considers the presence of an insulative plate around the stone, which gradually deteriorates over time and, subsequently, predisposes the stone to an infection and resultant abscess formation [3]. A literature review conducted on 91 patients by Brockman et al. identifies subhepatic abscesses as the most common abscesses (34.1 %), followed by subphrenic (15.9%) and ovarian abscesses (11.4% ). Other, less-common adverse outcomes include bowel obstruction and perforation (2.2%), peritonitis (3.3%), fistula formation (12.1%), ileus (2.2%), empyema, broncholithiasis, and cholelithoptysis through a peritoneo-pleuro-bronchial fistula [7-8].

The clinical presentation of an abdominal abscess is dynamic and mostly depends on the location of the abscess. Most features are non-specific and include low-grade fever, anorexia, weight loss, persistent abdominal pain, specific tenderness at the site of the abscess, and paralytic ileus. Laboratory evaluation may show leukocytosis, elevated erythrocyte sedimentation rate (ESR), and blood culture may reveal polymicrobial growth [2,9]. However, there have been cases of abdominal abscesses which are clinically silent and only identified following routine radiological investigations [10].

Diagnosis is challenging due to the unusual location of the abscess. It is additionally problematic in cases where there is no official documentation of a gallstone spillage [4]. Such outcomes underscore the significance of radiological imaging in the visualization of an abscess. Most common modalities include ultrasonic imaging, computed tomography (CT) scans, and magnetic resonance imaging (MRI). On ultrasonography, radiolucent gallstones are often visualized as hyper-echoic entities, which are bordered by aggregates of fluid or necrotic collections. The overlying calcification of the gallstones could render them radiopaque. Such stones can be visualized on CT imaging as hyperdense lesions while MRI scans could reveal a signal void, characterized by the presence of a blank space in a fluid-filled accumulate [1,11].

The management of spilled stones is multifaceted. The presence of an associated abscess warrants the utilization of antibiotics that target gastrointestinal microorganisms [2]. We suspected that our patient was septic and provided him with broad-spectrum antibiotic coverage. A lack of symptom resolution warranted the utilization of interventional modalities. A needle aspiration is necessary to verify the presence of an abscess, as imaging techniques may not be able to discriminate between other mass collections such as a biloma or a hematoma. The insertion of a pigtail catheter allows for the evacuation of purulent contents and helps in the abatement of sepsis. A CT-guided approach is usually preferred due to its ability to provide an accurate assessment of the extent of fluid pockets and the presence of underlying complications of inflammation such as adhesions. The tract formed by the sinus is then dilated after a few weeks and the abscess at the base of the canal is assessed via an abscessogram, which allows for the visualization of the cavity, the presence of gallstones, as well as any associated leading tracks. A nephroscope can be passed through the canal to assist in the subsequent stone evacuations. The size of the stone(s) guides further management. Stones less than 1 cm in size can be scooped via a basket; however, stones greater than 1 cm in size may warrant splintering to make evacuation easier. This can be accomplished by mechanical means or ultrasonic lithotripsy, but shattering stones could lead to the formation of smaller remnants, which may endure a resultant evacuation and instigate a secondary inflammatory response down the road. Following the procedure, a second abscessogram ensures that all gallstones have been evacuated and a secondary catheter is to be left in place and flushed regularly. Indications for its eventual removal include a cessation of primary clinical manifestations and an output of less than 10 mL per day [1,9].

We conducted a literature review of abdominal abscesses that developed after 10 (or more) years since a laparoscopic cholecystectomy utilizing PubMed. Our evaluation concluded that nine such cases have been previously reported, with our case being the 10th. The mean age of these patients was 59.6 years, ranging from 28 to 77 years. An overwhelming female predominance was observed (89%). Most common complaints included abdominal pain (78%), swelling (56%), and fever (33%) with the associated laboratory investigations, including leukocytosis (44%) and elevated CRP (22%). Commonly isolated organisms included *Enterococcus faecalis*, *Clostridium perfringens*, *Actinomyces israelii*, *Enterobacter aerogenes,* and *Escherichia coli*. The locations of abscesses varied considerably with each case, ranging from the pouch of Douglas to the subhepatic and perihepatic regions. Some cases were complicated by the extension of the abscesses to the lower back and the retroperitoneum. Most abscesses were treated with a combination of antibiotics and percutaneous drainage, but open laparotomies were performed in complicated cases. A majority of the patients (78%) have had a favorable clinical outcome to date (Table 1).

**Table 1:** Literature review of all the cases of abdominal abscesses that developed due to gallstone spillage after 10 (or more) years since a laparoscopic cholecystectomy, utilizing PubMed M: Male; F: Female; CT: Computed Tomography; MRI: Magnetic Resonance Imaging

PUBLICATION	AGE	SEX	TIME SINCE LAPAROSCOPIC CHOLECYSTECTOMY	CLINICAL PRESENTATION	SITE OF ABSCESS	DIAGNOSTIC MODALITY	TREATMENT	OUTCOME/RECOVERY
Christensen et al. [3]	53 years	F	17 years	Rectal pain, incomplete defecation, and feeling of lower abdominal heaviness	Recto-uterine pouch (pouch of Douglas)	CT scan	Transvaginal drainage of the abscess	Uneventful
Bartels et al. [5]	72 years	F	10 years	Intermittent, right upper quadrant abdominal and right-sided flank pain	Subhepatic	CT scan	Drainage of the abscess via exploratory laparotomy following failed ultrasound-guided drainage	Uneventful
Pottakkat et al. [6]	28 years	F	11 years	Painful and tender swelling in the right upper quadrant of the abdomen	Perihepatic	CT scan	Drainage of the abscess via an open exploration	Uneventful
Stupak et al. [12]	72 years	F	11 years	Fever, nausea, anorexia, and right upper quadrant abdominal pain	Subhepatic	CT scan	Percutaneous drainage of the abscess and intravenous clindamycin	Uneventful
Arishi et al. [13]	45 years	F	15 years	Colicky, central abdominal pain and swelling	Upper abdomen	CT scan	Surgical removal of the cyst	A wound infection 2 weeks postoperatively; managed with debridement and antibiotics
Başak et al. [14]	77 years	M	10 years	Vague abdominal pain	Three abscesses extending from the posterior aspect of the liver to the right flank	CT scan	Ultrasound-guided percutaneous drainage of the abdominal abscess. The flank abscess was managed with an open incision and drainage	Uneventful
Nugent et al. [15]	73 years	F	20 years	Pain in the lower back and the right gluteal region on walking; presence of a swelling over the right flank	Right paracolic gutter, communicating with an abscess in the superficial tissues of the right lower back	MRI scan of the abdomen and pelvis	Laparoscopic drainage of the abscess and a retrieval of the dropped gallstone	Formation of a peritoneo-cutaneous fistula, which was left to heal by secondary intention
Oh et al. [16]	73 years	F	15 years	Abrupt abdominal pain	Intra-abdominal abscess abutting the abdominal wall	CT scan	Ultrasound-guided aspiration followed by laparoscopic stone removal and abscess drainage	Uneventful
Hussain et al. [17]	43 years	F	10 years	Fever, tender swelling, and surrounding cellulitis in the right flank	Antero-lateral abdominal wall of the right lumbar region extending to the retroperitoneum	CT scan	Incision and drainage followed by secondary closure of the wound after 3 weeks	Uneventful

## Conclusions

Laparoscopic cholecystectomy is the gold standard for the treatment of gallstones. However, this new therapeutic modality brings new clinical challenges, such as gallstone spillage, which can lead to intra-abdominal abscesses. This may present acutely or even more than a decade later (as explained earlier). The clinical presentation is vague and presents a diagnostic challenge, which underscores the fact that treating physicians must harbor an adequate suspicion for this clinical entity, especially in patients with a previous history of laparoscopic cholecystectomy. Management focuses on a combination of antibiotic therapy and minimally invasive procedures that allow for abscess drainage, evacuation of gallstones, and subsequent resolution of symptoms.
